# A Case Series of Metastatic Malignant Gastrointestinal Neuroectodermal Tumors and Comprehensive Genomic Profiling Analysis of 20 Cases

**DOI:** 10.3390/curroncol29020109

**Published:** 2022-02-21

**Authors:** Taylor Kandler, Eliane Cortez, Lani Clinton, Amanda Hemmerich, Osama Ahmed, Ralph Wong, Taylor Forns, Andrea J. MacNeill, Trevor D. Hamilton, Mohammadali Khorasani, Xiaolan Feng

**Affiliations:** 1 Department of Medicine, University of British Columbia, Vancouver, BC V6T 1Z3, Canada; kandlertaylor@gmail.com; 2 Foundation Medicine, Inc., Cambridge, MA 02141, USA; ecortez@foundationmedicine.com; 3 Foundation Medicine, Inc., Morrisville, NC 27560, USA; lclinton@foundationmedicine.com (L.C.); ahemmerich@foundationmedicine.com (A.H.); 4 Department of Medical Oncology, Saskatoon Cancer Center, Saskatoon, SK S7N 4H4, Canada; osama.ahmed@saskcancer.ca; 5 Department of Medical Oncology, Cancer Care Manitoba, Winnipeg, MB R3E 0V9, Canada; rwong2@cancercare.mb.ca; 6 Department of Pathology, Duke University, Durham, NC 27710, USA; taylor.forns@duke.edu; 7 Department of Surgery, University of British Columbia, Vancouver, BC V5Z 1M9, Canada; andrea.macneill@bccancer.bc.ca (A.J.M.); trevor.hamilton@vch.ca (T.D.H.); kh.sohrab@gmail.com (M.K.); 8 Department of Medical Oncology, Tom Baker Cancer Center, Calgary, AB T2N 4N2, Canada; 9 Cumming School of Medicine, University of Calgary, Calgary, AB T2N 4N1, Canada

**Keywords:** malignant gastrointestinal neuroectodermal tumors (GNET), clear cell sarcoma (CCS), clear cell sarcoma-like tumor of the gastrointestinal tract (CCSTGT), EWSR1-ATF1 fusion, EWSR1-CREB1 fusion, comprehensive genomic profiling (CGP)

## Abstract

Malignant gastrointestinal neuroectodermal tumor (GNET) is an ultra-rare soft tissue sarcoma, therefore often misdiagnosed and has no available standard treatment. Here, we report 3 cases of metastatic GNET with variable clinical courses. Our small case series as well as extensive literature review, further support that GNET is a spectrum of diseases with variable inherent biology and prognosis. Surgical management in the setting of recurrent/metastatic disease may be appropriate for GNET with indolent nature. Response to systemic treatments including chemotherapy and targeted treatments is variable, likely related to heterogenous biology as well. Furthermore, we retrospectively identified 20 additional GNET cases from Foundation Medicine’s genomic database and expanded on their clinicopathological and genomic features. Comprehensive genomic profiling (CGP) with DNA and RNA sequencing of this cohort, in the course of clinical care, demonstrated recurrent EWSR1 chromosomal rearrangements and a sparsity of additional recurrent or driver genomic alterations. All cases had low tumor mutational burden (TMB) and were microsatellite stable.

## 1. Introduction

Malignant gastrointestinal neuroectodermal tumor (GNET) is an ultra-rare primary mesenchymal malignancy of the gastrointestinal tract, previously referred to as clear cell sarcoma-like tumor of the gastrointestinal tract (CCSLTGT) or “osteoclast-rich tumor of the gastrointestinal tract with features resembling clear cell sarcoma (CCS) of soft parts” [1,2]. The term GNET was introduced in 2012 by Stockman et al., whereby their 16-case series supported differentiating GNET from CCS as a distinctive tumor entity, rather than a variant [3]. As of December 2021, only 111 cases have been reported, lending challenge to the limited clinical, prognosticative, tumor staging or treatment and management information available [1]. Further, due to its rarity and similarities to other malignancies, GNET is often misdiagnosed and inappropriately treated [1,3]. Here, we present 3 GNET cases from 3 institutions in Canada and describe their clinical course, treatments, and outcomes. In addition, we performed extensive literature review with a specific focus on systemic treatments in both the adjuvant and metastatic setting as well as surgical management for recurrent/metastatic disease. Furthermore, we describe the molecular landscape and clinicopathological characteristics of a cohort of 20 GNET patients that underwent comprehensive genomic profiling (CGP) via next generation sequencing (NGS) performed in the context of routine clinical care.

## 2. Clinical Cases Presentation

### 2.1. Case 1

A 49-year-old Caucasian male presented with a 5-year history of intermittent episodes of abdominal cramping, non-bloody diarrhea, and weight loss. He was otherwise healthy except for a possible history of small fiber neuropathy present as intermittent burning sensation in his feet bilaterally, however nerve conduction studies were normal.

A colonoscopy was performed in September 2020, where a near-obstructing erythematous friable lesion was identified in the sigmoid colon. A biopsy was taken and revealed malignant spindle cells with occasional pseudoinclusions, moderate abundant cytoplasm and no obvious mitotic figures. Immunohistochemistry (IHC) staining was positive for S100, SOX-10, CD68 and negative for calretinin, melanA, HMB-45, chromogranin A, synaptophysin, CD117, CD34, muscle specific actin, and broad-spectrum keratin (AE1/3). Ki-67 was low, less than 1%. The initial diagnosis was made as benign schwannoma of the microcytic reticular type.

All staging imaging including computed tomography (CT) and magnetic resonance imaging (MRI) were negative. A laparoscopic sigmoid resection was performed in November 2020. There was no free fluid in the abdomen. A tumor was identified on the sigmoid colon that was firm, approximately 3–4 cm in length with a puckering of the serosa. Additionally, there were subtle 1–2 mm flat and soft white deposits on the pelvic peritoneum anteriorly and in the lower left quadrant. Laparoscopic sigmoid resection with low inferior mesenteric artery ligation was performed. In addition, representative sample of the peritoneal deposits were biopsied.

Pathology revealed a 2.2 cm, French grade 2, epithelioid and spindle tumor infiltrating through the full thickness of the bowel from ulcerated mucosa to serosal surface. The cellularity was moderate to high. Necrosis was not observed. There was no lymphovascular or perineural invasion. The mitotic figure was scanty with 1 in 10 high power fields. The tumor stroma was fibrous and myxoid. IHC revealed similar patterns as the previous biopsy. The Ki-67 in most areas was also less than 1% but very small foci demonstrated a higher proliferation rate, although less than 5% overall. The primary tumor margin was clear. All eleven lymph nodes were negative. However, the peritoneal biopsy revealed metastatic disease. Interestingly, an EWSR1 fusion was absent in this patient’s tumor as confirmed by 3 methods including Fluorescence In Situ Hybridization (FISH), NanoString and NGS.

Postoperatively, the patient denied any symptoms in relation to low volume residual peritoneal metastases. The postoperative CT scan as well as the positron emission tomography (PET) scan were negative. After extensive discussion, including at a multidisciplinary provincial sarcoma tumor board, a recommendation was made for short-interval close surveillance with no systemic treatment, in order to determine disease biology. If his disease did not exhibit rapid and widespread progression, it was felt that he could be considered for metastectomy. The most recent CT scan of the chest, abdomen and pelvis continue to demonstrate no radiologically apparent disease. He will proceed to diagnostic laparoscopy to assess burden and distribution of peritoneal disease, with a plan for complete resection if technically feasible.

### 2.2. Case 2

A 70-year-old Caucasian male without any significant medical comorbidities presented to a local hospital in March 2016 with a small bowel obstruction, for which he underwent laparoscopic-assisted small-bowel resection. Pathology revealed a malignant GNET, 3 × 2.8 × 1.3 cm with ulceration, invading the subserosa. IHC revealed S100, CD99 and CD56 positivity with a Ki-67 of 20%. Resection margins were clear, but there was lymphovascular invasion. All fifteen lymph nodes were negative for malignancy. No adjuvant chemotherapy was given. He was followed with annual colonoscopy and imaging studies. CT scan in September 2018 revealed a new 10 cm mass in the right lobe of the liver. MRI and ultrasound-guided biopsy on October 15, 2018, confirmed metastatic GNET with similar cytopathological and IHC features to the primary. FISH was negative for EWSR1 and ALK fusions.

He started on palliative chemotherapy with doxorubicin and olaratumab in December 2019 and received 3 cycles without measurable response. He was referred to a regional center for possible surgical resection. There he underwent portal vein embolization in January 2019 followed by right hemicolectomy and caudate resection in April 2019. Pathology was consistent with metastatic GNET. Five metastatic foci (3.6–10 cm) were completely excised. He is currently under surveillance and his last CT in June 2021 showed no evidence of recurrence.

### 2.3. Case 3

A 49-year-old Caucasian male presented to his primary care physician with a history of general malaise. He had a remote history of a thymic B-cell lymphoma treated with cyclophosphamide, doxorubicin, vincristine, and prednisone (CHOP) chemotherapy and chest radiation in Germany. He presented with iron deficiency anemia in January 2018 and was found to have an abnormally thickened distal small bowel with surrounding lymphadenopathy on CT scan. He underwent an ileo-colic resection which showed a poorly differentiated tumor involving the full thickness of the ileum with osteoclast-like giant cells. The tumor showed diffuse positivity for S100 and SOX-10; negative for HMB-45, melanA, microphthalmia transcription factor, synaptophysin, chromogranin, CD 117, DOG 1, pancytokeratin, desmin and myo D1. Ki-67 was not performed. Molecular cytogenetic studies confirmed the presence of an EWSR1-ATF1 fusion.

Adjuvant therapy was not given, and the patient agreed to undergo surveillance. Two years later, he presented with multiple hepatic and symptomatic osseous metastases in the spine. Liver biopsy confirmed metastatic GNET. After palliative spinal radiation treatment (RT) for which he benefited clinically with a significant reduction of bone pain, he received three cycles of palliative chemotherapy in the form of doxorubicin from January 2020 to April 2020 without any clinical response. Treatment was discontinued due to significant toxicities and intolerance. He declined further systemic therapy and required further palliative RT for progressive and symptomatic osseous metastases. He responded to RT symptomatically but unfortunately passed away in August 2021, 36 months after the original diagnosis and 15 months after discontinuing systemic therapy.

## 3. Clinicopathological Characterization and Comprehensive Genomic Profiling (CGP) of 20 Additional GNET Cases from Foundation Medicine

### 3.1. Pathology

#### 3.1.1. Methodology

The 20 cases of GNET were retrospectively identified from the database of a large, Clinical Laboratory Improvement Amendments (CLIA)-certified and College of American Pathologists (CAP)-accredited, reference molecular laboratory. All formalin-fixed paraffin-embedded (FFPE) material was prepared by the originating labs and sent to the reference lab for CGP testing. The Hematoxylin and Eosin (H&E) slides were either stained in house or externally, for review at the time of specimen submission for NGS testing. The subsequent H&E slides underwent whole-slide imaging prior to archiving or return to the external pathology lab. Retrospectively, all 20 cases were reviewed by two gastrointestinal malignancy-trained pathologists from the reference lab (L.C. and A.H.) using whole-slide imaging. The review was done independently before results were compared, and the reviewers were not aware of CGP results at the time of review. Additional information regarding IHC staining results and EWSR1 FISH results was extracted from the pathology reports provided by the outside institutions.

#### 3.1.2. Histomorphologic Features

**Figure 1:**
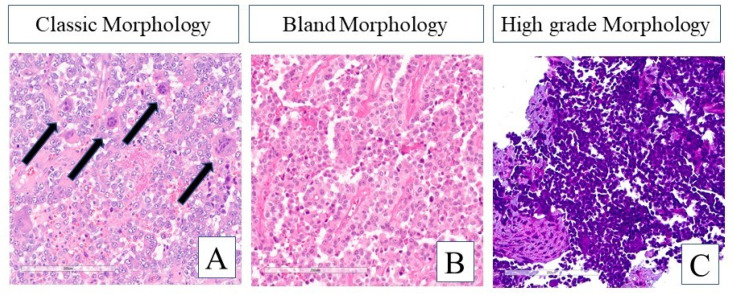
Range of histomorphologic features in GNET cases based on H&E staining. (**A**) Alveolar and pseudopapillary architecture with round to oval neoplastic cells containing vesicular nuclei with prominent nucleoli. Osteoclast-like multinucleated giant cells (black filled arrows) are present. (**B**) Similar architectural pattern to example in (**A**), however, the neoplastic cells are smaller and more bland in appearance as they have more evenly dispersed chromatin and lack prominent nucleoli. (**C**) Sheets of cells with very high N:C ratios containing nuclei with hyperchromatic chromatin. All photos taken at 200× magnification.

The 20 GNET cases demonstrated a wide range of morphologic features with respect to both architecture and cytology. The architectural patterns included those previously described such as solid, nested, alveolar, and pseudopapillary. Many of the cases also demonstrated cytologic features consistent with previous reports such as round to oval cells containing nuclei with vesicular chromatin and prominent nucleoli. The cytoplasm varied from pale eosinophilic to clear. Multi-nucleated osteoclast-like giant cells were identified in 4 of 20 cases (Figure 1A). However, cases were also observed where the cytologic features were markedly different from the classic GNET picture. A few cases demonstrated deceptively bland features with small, uniform nuclei and evenly dispersed chromatin without prominent nucleoli (Figure 1B). Moreover, other cases displayed features on the entirely opposite end of the spectrum and were characterized by high nucleus to cytoplasm (N:C) ratios, markedly hyperchromatic nuclear chromatic, and even focal areas of nuclear molding and crush artifact (Figure 1C).

#### 3.1.3. Immunohistochemistry and EWSR1 FISH

Utilizing the accompanying external pathology reports with samples for NGS testing, the following data on IHC and EWSR1 FISH results were extracted from such reports. Among 14 cases that were stained for S100, 14 were reported as positive (100%). SOX-10 staining was reportedly positive in 7 of 8 cases that were tested (88%). All 13 cases that were tested for melanoma markers were reported as negative (HMB-45, Melan-A, or melanoma cocktail). Immunohistochemistry for Ki-67 was performed on 8 cases (mean: 29%, range: 10–80%) (data not shown). EWSR1 FISH translocation was reported as positive in 11/11 cases in which it was performed (100%) (Table 1).

### 3.2. Genomic Profiling Analysis

#### 3.2.1. Methodology

CGP using next-generation sequencing was performed on hybridization-captured, adapter ligation-based libraries using DNA and RNA extracted from formalin-fixed paraffin-embedded (FFPE) tumor in a CLIA- and CAP-certified laboratory (Foundation Medicine, Inc. Cambridge, MA, USA). All samples forwarded for DNA and RNA extraction contained a minimum of 20% tumor cells. The samples were assayed using adapter-ligation and hybrid capture next-generation sequencing for all coding exons from up to 406 cancer related genes, plus select introns from up to 31 genes frequently rearranged in cancer. Patient samples were fully sequenced and evaluated for genomic alterations including base substitutions, insertions, deletions, copy number alterations (amplifications and homozygous deletions), and select gene fusions/rearrangements, as previously described [4,5]. The bioinformatics processes used in this study included Bayesian algorithms to detect base substitutions, local assembly algorithms to detect short insertions and deletions, a comparison with process-matched normal control samples to detect gene copy number alterations and an analysis of chimeric read pairs to identify gene fusions as previously described. To help visualize the sequencing data results, an oncoprint plot was generated with online tools from cBioPortal [6,7].

#### 3.2.2. Genomic Findings

**Figure 2:**
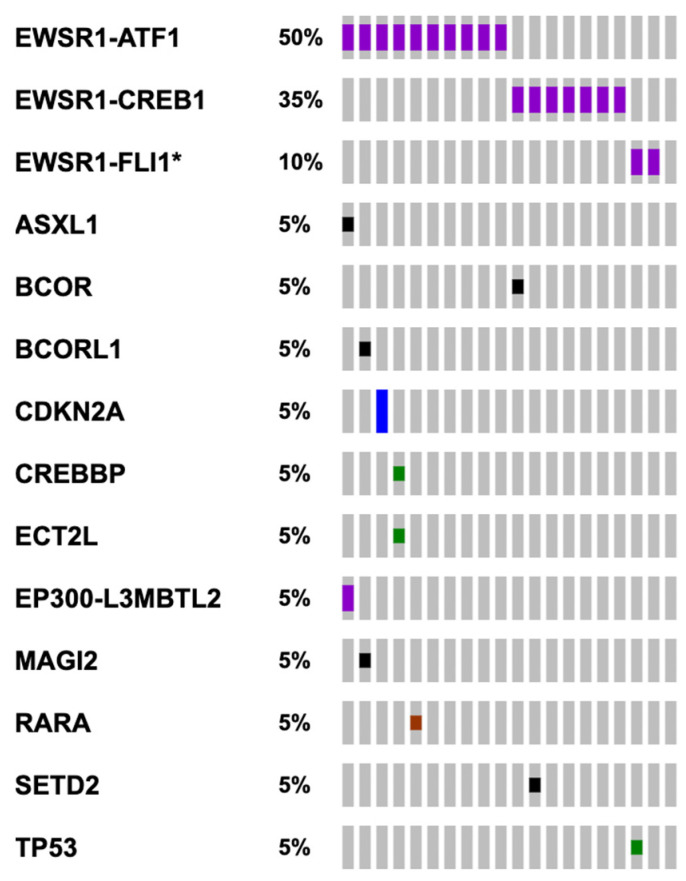
Mutational landscape of 20 gastrointestinal neuroectodermal tumors (GNET). Each column represents one patient/case. Purple: Gene fusions/Rearrangements. Green: missense mutation. Red: indel mutation. Black: Truncating Mutation. Blue: copy number deletion. Variants of unknown significance were excluded. * EWSR1-FLI1 cases are discussed in more detail in Section 4.7.

**Figure 3:**
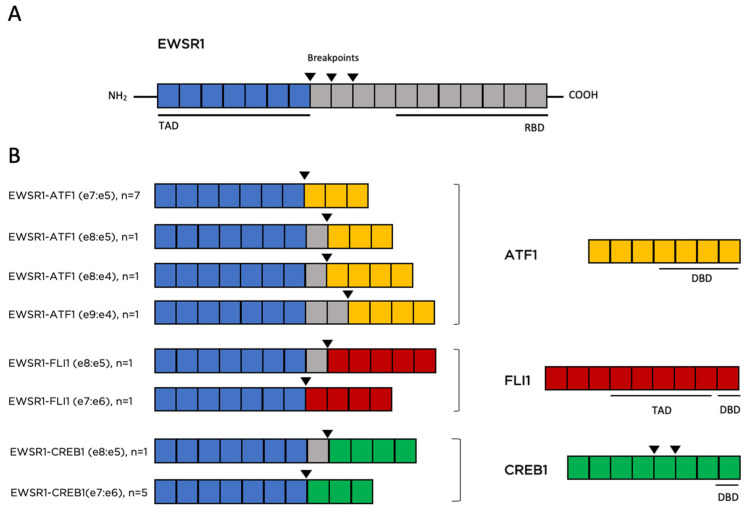
Schematic representation of EWSR1 fusions identified in a cohort of 20 GNET cases profiled by next generation sequencing. (**A**) Schematic diagram showing EWSR1 exons and corresponding protein domains. (**B**) Left: predicted gene fusions. Right: schematic of partner genes (exons) and corresponding protein domains. All cases fuse the N-terminal transcriptional activation domain (TAD) of EWSR1 to a partner gene with a DNA binding domain (DBD). Black inverted triangles indicate the fusion breakpoints. Gene fusions are not drawn to scale. RDB: RNA binding domain.

The 20 cases submitted to Foundation Medicine where sequenced using either FoundationOne or FoundationOne CDx (DNA sequencing only, *n* = 8) to detect all classes of genomic alterations including substitutions, indels, gene fusions/rearrangements and copy number alterations, or FoundationOne Heme (DNA and RNA sequencing, *n* = 12). In addition to the DNA sequencing, the RNA sequencing component of FoundationOne Heme only allows detection of fusion transcripts but is not used for analysis of RNA expression. Of the 20 samples, 19 harbored EWSR1 chromosomal translocation events at the DNA and/or RNA level (95%). These translocations included t(12;22)(q13;q12), t(2;22)(q34;q12) and t(11;22)(q24;q12), leading to EWSR1-ATF1 (*n* = 10), EWSR1-CREB1 (*n* = 6) and EWSR1-FLI1 (*n* = 2) fusions respectively (Figure 2 and Figure 3). Common breakpoints in EWSR1 were found in introns 7 and 8 (Figure 3) similar to EWSR1 fusions found in other malignancies. Additional genomic alterations including short variants (base substitutions, insertions, and deletions), and copy number alterations were infrequently detected and only included CDKN2A/B homozygous copy number loss, and short variants in ASXL1, BCOR, BCORL1, CREBBP, ECT2L, MAGI2, TP53, SETD2 and RARA, each occurring in one case only (Figure 2). For one case with no EWSR1 fusion identified by NGS, an EWSR1 chromosomal rearrangement was reported by FISH analysis. This specimen was sequenced using an older bait set that did not cover the entire EWSR1 gene and for that reason a fusion could have been missed. Another case harbored a CREB1-EWSR1 rearrangement, but the reciprocal fusion (EWSR1-CREB1) was not detected. Again, this is likely because EWSR1 was not fully baited with older sequencing bait sets. For 11 of 19 cases with EWSR1 fusions no additional genomic alterations were identified from DNA- and RNA-based sequencing (Figure 2).

Biomarkers of response to immune checkpoint blockade were also absent. These tumors have a low tumor mutational burden (TMB) (median 1.25 mutations/megabase), and microsatellite instability was not detected in any of the 19 cases where it was analyzed (Table S1).

## 4. Literature Review and Discussion

### 4.1. Clinical Presentation Is Variable and Nonspecific

The median age at diagnosis of GNET is 36 years, however GNET patients present with a wide range of age distribution and no gender predilection [1,2,3,8]. Patients commonly present with gastrointestinal symptoms including abdominal discomfort/pain, distension, obstruction, ascites, pelvic effusions, or abdominal masses clinically or on imaging [1,2,3,8]. Nonspecific symptoms such as anorexia, anemia, weight loss, high-grade fever, and weakness have also been described in case reports [1,3,9]. This variability of clinical presentation aligns with our 3 clinical cases presented here. GNET originate primarily in the small intestine, and less commonly in the stomach, large intestine, ileocecal junction, anal canal, and lower esophagus [1,3,8]. Metastatic disease is not uncommonly established at the time of diagnosis, found in 29% of cases in a small case series [1]. Our first case presented with synchronous metastatic disease and the other two cases presented with local disease initially and recurred metastatically.

### 4.2. Accurate Pathological Diagnosis Remains a Significant Challenge

Histologically, these tumors have primitive epithelioid, oval or spindle tumors cells, and osteoclast-type giant cells are commonly described. Given the varying histology, in particular prominent epithelioid or spindle cell components, these tumors can be misdiagnosed for a variety of other diagnoses including a poorly differentiated carcinoma, such as a sarcomatoid carcinoma [1]. A thorough IHC panel including cytokeratins, S100 and SOX-10 can aid in differentiating the tumor as a GNET. GNET are negative for cytokeratins while carcinomas are positive [1]. The IHC hallmark of GNET tumors is positivity for S100 and SOX-10 proteins and lack of melanocytic-specific markers, such as HMB-45 and melanA. This IHC profile is suggestive of primitive neural phenotype [2,3,10]. Stockman et al. suggests that GNET tumors “may arise from an autonomic nervous system-related primitive cell of neural crest derivation that shows a neural line of differentiation with complete absence of melanocytic features”, supporting the Antonescu et al. theory of GNET arising from neuroectodermal precursor cells with lost differentiating potential [3,10]. Additionally, it is notable that mitotic rate and Ki-67 of reported GNET cases spanned a wide range between 1–30 (most cases around 10–12) of 10 High Power Field and 5–90% (most cases around 20%) respectively [1,11,12,13,14,15,16,17,18,19,20,21,22,23,24,25]. This observation is consistent with our reported cases.

Molecularly, GNET harbor a hallmark t(12;22)(q13;q12) and t(2;22)(q34;q12) chromosomal rearrangement, resulting in chimeric fusion proteins EWSR1-ATF1 and EWSR1-CREB1, respectively. Stockman et al. found EWSR1 rearrangements in 12 out of 14 cases (86%). Of these, EWSR1-ATF1 and EWSR1-CREB1 rearrangements were found in 6 and 3 cases respectively. Chang et al. found EWSR1 rearrangements in 93.3% of cases [1,3]. Both studies used FISH to detect EWSR1 rearrangements. Therefore, EWSR1 rearrangement, albeit occurring frequently, is not an absolute diagnostic criterion for GNET. One of our 3 clinical cases (case 1) was negative for EWSR1 fusion confirmed by FISH, NanoString and NGS. Additionally, EWSR1 rearrangements are not unique to GNET but are also found in many other mesenchymal and non-mesenchymal malignancies such as CCS, Ewing sarcoma, desmoplastic small round cell tumor, myxoid round cell liposarcoma, myoepithelial carcinoma, epithelioid mesothelioma as well as “benign” tumors such as angiomatoid fibrous histiocytoma tumor [23,26]. It is generally thought that EWSR1 fusions with various partner genes occurs early and serves as a molecular driver in the process of oncogenesis [23].

### 4.3. Initially Thought to Be a Highly Aggressive Tumor, Prognosis of GNET Is Variable

Variable prognoses of GNET have been reported in the literature. Some report that GNET is a highly aggressive tumor with high recurrence rates and dismal prognosis [3,19,27], while others report somewhat better outcomes [1,22,28,29,30]. Li et al. did a comprehensive analysis of clinical outcomes of 96 published cases to date (2020) and revealed that overall survival (OS) ranged from 0.69 to 161 months, with a median of 61 months [20]. However, 8 patients had prolonged survival of over 5 years. Disease-free survival (DFS) ranged from 1 to 109 months, with a median of 10.0 months; the median time to first metastasis was 12 months and just over 80% of patients developed metastatic disease within 2 years [20]. It remains unknown if better clinical outcomes for some GNET patients are the result of their indolent disease biology or of more aggressive treatments such as repeated radical surgery/metastectomy and/or systemic chemotherapy/targeted treatments. We believe that GNET, although ultra-rare, is a biologically heterogenous disease, similar to all cancer types. As shown in our 3 clinical cases, GNET may be a spectrum of diseases, ranging from indolent to highly aggressive. This biological heterogeneity is further reflected by the distinct histomorphologic features of the additional 20 cases examined in our study (Figure 1). It is important to characterize the disease biology to guide clinicians in tailoring individual treatment strategies.

### 4.4. Surgery Remains a Main Treatment Modality in Both Localized and Metastatic Settings

Surgery remains the mainstay of treatment for localized GNET. Upon disease recurrence, repeated radical surgical resection appears to contribute to long DFS [29,31]. Song et al. reported a 23-year-old man who presented with a locally recurrent esophageal GNET one year after initial esophageal enucleation of a primary tumor and one round of RT. Upon tumor progression, after less than one year of surveillance as per the patient’s preference, he underwent re-resection of progressive tumor using the Ivor-Lewis procedure successfully and remained well with no evidence of disease locally or distantly in the 2-year follow up period [31]. Sivaubramaniam et al. reported a 46-year-old woman who presented with recurrent metastatic GNET with locoregional lymphadenopathy 17 months after initial distal partial gastrectomy. She underwent laparotomy and excision of perigastric and peripancreatic lymph nodes. She remains disease free at the time that the manuscript was written, however follow-up time is unknown [32]. In the setting of distant metastasis, especially in the liver, metastectomy, sometimes in conjunction with chemotherapy, was performed in a number of reported cases which may contribute to prolonged survival in these cases [18,29,33,34]. To our knowledge, there is no literature to inform the role of cytoreductive surgery and possibly intraperitoneal chemotherapy (CRS/HIPEC) for GNET with peritoneal metastases. CRS/HIPEC has been extensively studied in colon and other common cancers where prognostication tools to select appropriate candidates have been developed [35,36,37]. This technique will be considered for our case 1 if he is found on laparoscopy to have resectable, low to moderate volume peritoneal disease.

### 4.5. Efficacy of Chemotherapy and Targeted Therapies Remains Largely Unknown and Appears to Be Variable

#### 4.5.1. Adjuvant Chemotherapy for Localized Disease

The efficacy of neoadjuvant or adjuvant chemotherapy is unknown for localized GNET. There are only scarce case reports in which adjuvant chemotherapy was utilized (Table 2), likely because adjuvant chemotherapy is generally not recommended in resected non-extremity soft tissue sarcoma [38]. In addition, in the few existing case reports, the outcome was not reported after neoadjuvant/adjuvant chemotherapy as patients were lost to follow up [14,39]. Singh et al. reported a 61-year-old African American patient with a resected GNET of the right colon harboring a EWSR1-CREB1 fusion that experienced a DFS of 7 years before metastatic recurrence after adjuvant platinum and etoposide chemotherapy [22]. Other adjuvant sarcoma-based systemic treatments such as doxorubicin or epirubicin and ifosfamide, doxorubicin and dacarbazine, dacarbazine and cisplatin, vincristine, doxorubine and cyclophosphamide have been used in some case reports with variable outcomes and follow up [1,14,15,18,25,40,41]. Some reports did not specify the type of adjuvant chemotherapy [2,13,24,42,43].

#### 4.5.2. Systemic Treatments for Metastatic Disease

Similarly to localized GNET, there are limited reports on the efficacy of systemic treatments for advanced/metastatic GNET as surgery has been the sole therapeutic approach in most cases. We performed an extensive literature search and summarized the cases where systemic treatments were attempted (Table 2). Briefly, metastatic GNET has generally variable response to standard sarcoma-based systemic chemotherapy regimens. Although most cases reported poor chemotherapy sensitivity [1,13,16,24,33,34,41,42,43], which is in keeping with the morphologically similar entity CCS [52], a few cases reported partial response (PR) or stable disease (SD) [1,8,46,50]. Interestingly, gastrointestinal cancer-based chemotherapy such as oxaliplatin, irinotecan and paclitaxel were also explored in one patient who achieved a short-lived clinical benefit [1]. It is notable that treatment response to chemotherapy is not necessarily related to higher Ki-67 or mitotic rate [1,8]. Two of our cases (case 2 and 3) demonstrated a degree of chemo-resistance, one of which had a relatively high Ki-67 of 20%.

Oral targeted therapies such as mammalian target of rapamycin (mTOR) inhibitors and multityrosine kinase inhibitors (TKIs) have been explored in a few cases of GNET and some appeared to have some activity. One patient was treated with everolimus for 5 months followed by sunitinib for 3 months with mixed but short-lived response [22]. Sunitinib was also used in another case without any clinical benefit [1]. Subbiah et al. reported a 27-year-old female patient diagnosed with metastatic GNET harboring EWSR1-CREB1 who exhibited a durable near-complete response to crizotinib and pazopanib for one and a half years on a clinical trial [49]. It is unknown if this remarkable response was contributed to by either crizotinib or pazopanib or both. It is also somewhat unexpected as EWSR1 fusions are not known to be predictive biomarkers for any targeted therapy such as TKIs. The authors proposed that this response may be related to receptor tyrosine kinase c-MET inhibition by promiscuous TKIs since the c-MET pathway is known to be upregulated by EWSR1-ATF1 or EWSR1-CREB1 fusions through activation of the melanocyte transcription factor (MITF) [49]. Crizotinib has been shown to provide clinical benefit for locally advanced or metastatic c-MET positive CCS [53]. Pazopanib alone was also attempted in other cases, where some patients achieved clinical benefit [1,32,51], but others did not [1,22]. Other TKIs such as apatinib and anlotinib were attempted, leading to PR and SD however these lasted less than 6 months in a few patients [1]. BRAF mutation, although absent in most GNET, was reported in one case (the genomic variant was not specified). This patient achieved PR to BRAF inhibitor dabrafenib in combination with trametinib after 3 months, but response was short-lived, for less than 6 months [43]. Immunotherapy was also attempted in a few cases alone or in combination with TKIs, however no clinical benefit was observed [1,43].

### 4.6. Radiotherapy Potentially Beneficial in the Metastatic Setting

It is not known whether GNET is sensitive to RT. In the few case reports where RT was utilized in the adjuvant setting, GNET were localized to the esophagus [28,31,47] and the results were variable [28,31,47]. Palliative RT is frequently used for symptom control in the metastatic setting regardless of cancer type. Our patient from case 3 benefited from palliative RT with symptomatic improvement of his progressive bone disease. Therefore, this approach could be considered for patients with symptomatic GNET, especially with bone metastasis.

### 4.7. EWSR1 Translocations Are the Most Recurrent Genomic Alteration and Other Potentially Targetable Genomic Alterations Are Rarely Identified

To the best of our knowledge, our study represents the first report of CGP of GNET via targeted DNA- and RNA-based NGS. Our aim was to expand on the genomic characterization of GNET to identify additional molecular alterations with potential therapeutic implications. Based on CGP analysis of 20 cases, we validated EWSR1 fusions as the most prevalent molecular event in GNET and provided additional granularity into different fusion variants and partner genes with variable breakpoints present in these tumors.

Interestingly, two cases in our cohort harbored a EWSR1-FLI1 fusion that is a known hallmark in Ewing sarcoma [23]. A few studies have reported FLI1 immunoreactivity in the context of GNET, but it is unclear whether a GNET diagnosis was confirmed or whether a EWSR1-FLI1 fusion was present in these cases. One study reported a case of GNET tumor which immunophenotypically unusually expressed FLI1, occurring in a 29-year-old man with a previous medical history of Ewing sarcoma [46]. In another case report, FLI1 immunoreactivity was reported to be strongly positive in one patient. This patient was diagnosed and treated for Ewing sarcoma with complete remission 24 years prior to the appearance of GNET [9]. Another report of GNET described a case positive for CD56, CD99, FLI1, and synaptophysin, and negative for chromogranin, TTF-1, SMA, desmin, CD34, EMA, pan-cytokeratin, and lymphoid markers; there were no reportable IHC results for S100 or SOX-10 and no reportable EWSR1 rearrangement precluding a final diagnosis of GNET [54]. Based on pathology assessment of the two EWSR1-FLI1 positive cases in our cohort, one did not have any reported IHC information, and the other was positive for S100 (as well as CD99 and vimentin but these are nonspecific markers and can be seen in GNET) but negative for SOX-10. Neither case had Ki-67 IHC performed. Histologically, both cases have small blue cells reminiscent of primitive oval epithelioid cells. No osteoclast-type giant cells were identified in either the submitted sample for testing or described in the pathology reports. These cases were classified as GNET based on the provided outside diagnosis. It is difficult to determine based on the provided information for testing if these cases were misdiagnoses or represent a novel fusion in GNET.

Besides EWSR1 gene rearrangements, additional genomic alterations were infrequently detected in the 20 GNET cases, limiting the options for targeted therapy for these patients. Despite these findings, a few patients from previous case reports who responded well to multi-targeted TKIs with either PR or durable SD may suggest that other types of molecular alterations (at the transcriptomic and/or proteomic level) underlying GNET tumorigenesis and response to targeted therapy were not appreciated in the current study. For example, high expression of genes within the vascular epidermal growth factor (VEGF) or mitogen-activated protein kinase (MAPK) pathways cannot be detected in the CGP analysis performed by Foundation Medicine as these assays include only DNA- and RNA-based NGS for detection of all classes of genomic alterations and select fusion transcripts respectively. As shown in this and other studies, different genes, mainly encoding for transcriptional regulators, can translocate with EWSR1 in GNET. The resulting chimeric EWSR1 fusions have been shown to interfere with different signaling pathways crucial for cell growth, differentiation, and proliferation. These interactions are often responsible for the pathogenesis of soft tissue tumors [55] and can potentially account for different responses to targeted therapies including diverse TKIs. Future research should focus on genomic alterations on RNA and protein levels as well as further functional assays that may help elucidate the molecular mechanisms of GNET progression and response to treatments.

Additionally, the varied reported responses to TKIs might simply reflect the lack of knowledge of molecular mechanisms of action of promiscuous TKIs in GNET and many other types of sarcomas in general. Nevertheless, based on genomic analysis only, the lack of other recurrent driver alterations suggests that EWSR1 fusions are the main drivers of GNET.

## 5. Conclusions

Even though GNET is an ultra-rare type of sarcoma, it may represent a spectrum of diseases with distinct histomorphology, clinical presentation and outcome as well as treatment response to various systemic therapeutics. EWSR1 gene rearrangement is the hallmark feature and molecular driver of GNET, but not an absolute diagnostic criterion. Evidence from this and other reports have emphasized that the accurate diagnosis of GNET requires extensive pathological expertise. It is essential to recognize the level of heterogeneity both pathologically and clinically, which not only might facilitate the correct diagnosis but is also paramount to guide appropriate clinical management. Our experience and the available but still scarce literature on GNET, suggest that surgical management in the setting of recurrent/metastatic disease may be appropriate for cases demonstrating indolent biology. Further comprehensive genomic, transcriptomic, and proteomic analysis, especially for unusual responders to different lines of treatment, may shed light on the molecular mechanisms of action of existing and novel therapeutic interventions for GNET.

## Figures and Tables

**Table 1:** Patient characteristics including prior immunohistochemistry (IHC) and EWSR1 FISH results.

Characteristic	Result
Median age (range), years	37.5 (15–64)
Sex	13 female, 7 male
Specimen site	
Small intestine	9
Liver	5
Soft tissue	4
Appendix	1
Stomach	1
Immunohistochemistry	
S100	14/14 positive
SOX-10	7/8 positive
HMB-45/Melan-A/melanoma cocktail	0/13 positive
EWSR1 FISH	11/11 positive for translocation

**Table 2:** Comprehensive literature review of adjuvant and metastatic treatments and clinical outcomes for published GNET case reports/case series.

Reference	Case No.	Age/Gender	Local/Recurrent/Metastatic Disease	Adjuvant/Metastatic Treatment (All Patients Underwent Primary Tumor Resection)	Clinical Outcome
Alyousef et al., 2017 [9]	1	18 M	Local recurrent	No	DFS 36 mos, DD 12 mos after local recurrence
Antonescu et al., 2006 [10]	3	81 F	De novo metastatic	Metastatic: Liver metastectomy and peritoneal implants removal (NA)	NA
		42 F	Local	NA	NA
		42 F	De novo metastatic	NA	NA
Boland et al., 2016 [44]	1	46 F	Local	NA	NA
Chang et al., 2020 [1]	19	29 F	De novo metastatic	NA	NA
		44 M	De novo metastatic	No	OS 61 mos
		41 M	Recurrent metastatic	Adjuvant: no Metastatic: radiofrequency ablation of liver metastasis (CR), no systemic treatment	DFS 8 mos NED F/U 63 mos
		42 M	Local	Adjuvant: dacarbazine + cisplatin	NED F/U 46 mos
		51 F	Recurrent metastatic	NA	DFS 11 mos OS 36 mos
		30 F	Recurrent metastatic	Adjuvant: no Metastatic: 1st line: sunitinib (stopped due to SE), 2nd line: anlotinib (PR for 6 mos)	DFS 14 mos AD F/U 43 mos
		48 F	NA	NA	NA
		42 F	Local	No	NED F/U 3 mos
		41 F	Recurrent metastatic	NA	DFS 4 mos AD F/U NA
		56 F	De novo metastatic	Metastatic: Liver metastectomy, no systemic treatment	NED F/U 33 mos
		30 F	NA	NA	NA
		59 M	Local	No	NED F/U 29 mos
		27 F	Recurrent Metastatic	Adjuvant: no Metastatic: apatinib (PR for 4 mos stopped due to SE)	DFS 13 mos AD F/U 26 mos
		47 M	Recurrent metastatic	Adjuvant: no Metastatic: 1st line: pazopanib + pembrolizumab (stopped due to SE), 2nd line: lenvatinib + pembrolizumab (PD), 3rd line: epirubicin + ifosfamide (SD for 3 mos)	DFS 27 mos AD F/U 48 mos
		36 M	Local	Adjuvant: Vincristine amide + Adriamycin + cyclophosphamide	NED F/U 3 mos
		40 F	Local	Adjuvant: Adriamycin + dacarbazine	NED F/U 8 mos
		41 F	Recurrent Metastatic	Adjuvant: no Metastatic: 1st line: oxaliplatin (PD), 2nd line: irinotecan (PD), 3rd line: paclitaxel (PD), apatinib (SD for 5 mos)	DFS 17 mos AD F/U 29 mos
		64 M	Local	Adjuvant: adriamycin + ifosfomaide	NED F/U 14 mos
		55 M	NA	NA	NA
Comunoglu et al. 2015 [45]	1	9 M	Local	Adjuvant: chemotherapy (NA)	NED F/U 12 mos
Damle et al., 2021 [8]	1	56 M	*De Novo* Metastatic	Metastatic: VAC/IE (PR after 3 cycles then PR/SD additional 11 cycles)	AD F/U 3 mos
Friedrichs et al., 2005 [39]	1	41 M	Recurrent Metastatic	Adjuvant: no Metastatic: ifosfamide, vincristine, actinomycin D, followed by ifosfamide and epirubicin (NA)	DFS: 6 mos AD F/U NA
Gadde et al., 2021 [13]	1	36 F	De novo metastatic	Metastatic: liver metastectomy, dacarbazine + gemcitabine (PD)	OS 4 mos
Harshavardhini et al., 2021 [14]	1	33 M	Local	Adjuvant: vincristine, etoposide, adriamycin, cyclophosphamide, mesna and ifosfamide	NED F/U 8 mos
Huang et al., 2019 [15]	1	30 F	Local	Adjuvant: ifosfamide + epirubicin	NED F/U 6 mos
Huang et al. 2020 [16]	4	45 F	Local	No	NED F/U 41 mos
		34 F	Local	No	NED F/U 17 mos
		81 M	Local with residual disease	No	AD F/U 8 mos
		68 M	De novo metastatic	Metastatic: chemotherapy (PD)	OS 5 mos
Insabato et al., 2015 [46]	1	29 M	Recurrent metastatic	Adjuvant: no Metastatic: 1st line: IE (PR/SD for 8 mos) 2nd line: ifosfamide alone (SD)	DFS 36 mos AD F/U 39 mos
Kansal & Rao, 2017 [17]	1	55 F	NA	NA	NA
Keditsu et al., 2017 [18]	1	37 F	De novo metastatic	Metastatic: liver metastectomy followed by pseudoadjuvant VAC/IE	NED F/U 36 mos
Kim et al. [47]	1	21 M	Local	Adjuvant: RT	NED F/U 5 mos
Kong et al., 2014 [19]	1	17 M	NA	NA	NA
Li et al., 2020 [48]	2	17 M	Local	No	NED F/U 10 mos
		62 M	De novo metastatic	No	AD F/U 6 mos
Libertini et al., 2018 [34]	6	59 F	Recurrent metastatic	Adjuvant: no Metastatic: dacarbazine (PD)	DFS 11 mos OS 18 mos
		28 F	Local recurrent	Resection for local recurrence, no systemic treatment	DFS 109 mos NED F/U 161 mos
		27 F	Recurrent metastatic	NA	DFS 2 mos OS 4 mos
		33 M	Recurrent metastatic	Adjuvant: no Metastatic: no	DFS 2 mos OS 8 mos
		48 M	Recurrent local/metastatic	NA	DD F/U NA
		27 M	De novo metastatic	Metastatic: metastectomy (peritoneal resection), no systemic treatment	NED F/U 2 mos
Lyle et al., 2008 [42]	7	46 M	Local	Adjuvant: chemotherapy (NA)	NED F/U 7 mos
		62 M	De novo metastatic	Metastatic: chemotherapy (NA)	OS 12 mos
		49 M	De novo metastatic	No	OS 2 mos
		60 F	De novo metastatic	NA	NA
		29 F	De novo metastatic	NA	NA
		60 M	De novo metastatic	Metastatic: chemotherapy (NA)	OS 28 mos
		55 F	NA	NA	NA
Okada et al., 2020 [29]	1	38 F	De novo metastatic	Metastatic: liver Metastectomy, no systemic treatment	NED F/U 36 mos
Shah et al., 2015 [28]	1	28 F	Recurrent metastatic	Adjuvant: RT, no systemic treatment Metastatic: IL-2 (PD), anti-CTLA4 (SD for 10 mos), anti-PD-L1 (SD for 7 mos), IL-15 (PD)	DFS 48 mos AD F/U 72 mos
Singh et al., 2020 [22]	1	61 M	Recurrent metastatic	Adjuvant: cisplatin+etoposide Metastatic: 1st line: capecitabine+temozolomide (PD), 2nd line: everolimus (SD for 5 mos), 3rd line: pazopanib (PD), 4th line: sunitinib (SD for 3 mos)	DFS 84 mos OS 13–15 mos
Sivasubramaniam et al., 2021 [32]	1	46 F	Recurrent metastatic	Adjuvant: no Metastatic: metastectomy (intraabdominal lymph nodes) followed by pseudoadjuvant pazopanib	DFS 17 mos NED F/U NA
Stockman et al., 2012 [3]	16	30 F	NA	NA	AD F/U 21 mos
		35 M	NA	NA	OS 18 mos
		33 M	NA	NA	AD F/U 1.5 mos
		50 F	NA	NA	AD F/U 24 mos
		20 F	NA	NA	NED F/U 20 mos
		52 M	NA	NA	OS 22 mos
		46 M	NA	NA	NA
		34 F	NA	NA	OS 19 mos
		37 F	NA	NA	NA
		77 F	NA	NA	OS 106 mos
		31 M	NA	NA	OS 3 mos
		17 M	NA	NA	NA
		30 M	NA	NA	AD F/U 36 mos
		60 F	NA	NA	NED F/U 41 mos
		56 M	NA	NA	NA
		28 F	NA	NA	OS 23 mos
Song et al., 2018 [31]	1	23 M	Local recurrence	Adjuvant: RT Local recurrence: surgical resection, no systemic treatment	DFS 12 mos NED F/U 24 mos
Subbiah et al., 2016 [49]	1	27 F	De novo metastatic	Metastatic: metastectomy (liver and others), cryotherapy, palliative RT, crizotinib + pazopanib (PR for 1.5 yrs)	AD F/U 2.8yrs
Wang et al., 2020 [50]	1	30 F	De novo metastatic	Metastatic: chemotherapy (NA, PR)	AD F/U 6 mos
Washimi et al., 2017 [40]	1	32 F	Recurrent metastatic	Adjuvant: ifosfamide + adriamycin Metastatic: NA	DFS 38 mos AD F/U NA
Wolak et al., 2018 [33]	1	12 M	Recurrent metastatic	Adjuvant: vincristine + adriamycin + ifosfamide + dactinomycin, followed by carboplatin + epirubicin + vincristine + actinomycin D + ifosfamide + etoposide Metastatic: thermal ablation (PD), liver metastectomy (PD), 1st line: carboplatin, epirubicin, vincristine, actinomycin D, ifosfamide and etoposide (PD), 2nd line: pazopanib (PD)	DFS 8 weeks OS 18 mos
Yagi et al., 2020 [43]	1	66 F	De novo metastatic	Metastatic: 1st line: dabrafenib + trametinib (PR for 3 mos), 2nd line: nivolumab + ipilimumab (PD)	OS 21 mos
Yegen et al., 2015 [41]	1	25 F	De novo metastatic	Metastatic: Liver Metastectomy followed by chemotherapy (NA, PD)	AD F/U 47 mos
Zambrano et al., 2003 [2]	6	15 F	De novo metastatic	Metastatic: chemotherapy (NA, PD)	OS 16 mos
		21 F	De novo metastatic	Metastatic chemotherapy (NA, PD)	OS 12 mos
		35 F	Recurrent metastatic	Adjuvant: No Metastatic: NA	DFS 12 mos AD F/U NA
		37 F	Local	NA	NA
		13 M	Recurrent local/metastatic	Adjuvant: chemotherapy (NA) Recurrence: total gastrectomy for local recurrence followed by pseudoadjuvant chemotherapy (NA)	DFS 7 mos AD F/U 5 mos
		32 M	Local	NA	NA
Zhan et al., 2019 [51]	1	33 F	Recurrent metastatic	Adjuvant: chemotherapy (NA) Metastatic: metastectomy (mesentery), sunitinib (SD for 12 mos)	DFS 14 mos AD F/U 12 mos
Zhao et al., 2014 [25]	1	33 F	Local	Adjuvant: ifosfamide + epirubicin	NED F/U NA
Zhao et al., 2017 [24]	2	57 M	local	No	NED F/U 16 mos
		24 M	De novo metastatic	Metastatic: 1st line: paclitaxel + gemcitabine (PD), 2nd line: vinorelbine + gemcitabine (PD), 3rd line: apatinib (SD for 2 mos), 4th line: apatinib+temozolomide (SD for 3–4 mos)	AD F/U 55 mos

DFS = Disease Free Survival, CR = Complete Response, PR = Partial Response, SD = Stable Disease, DD = Died of Disease, NED = No Evidence of Disease, NA = Not Available, OS = Overall Survival, AD = Alive with Disease, F/U = Follow Up, SE = side effects, mos = months, yrs = years, RT = Radiation Treatment, VAC/IE = vincristine + adriamycin (actinomycin D) + cyclophosphamide/ifosfamide + epirubicin, IL = Interleukin, CTLA4 = T-lymphocyte-associated protein 4, PD1 = anti-programmed cell death protein 1.

## Data Availability

The data presented in this study are available on request from the corresponding author. The data are not publicly available due to protection of patient privacy.

## Supplementary Materials

The following supporting information can be downloaded at: https://www.mdpi.com/article/10.3390/curroncol29020109/s1, Table S1: Detailed information of the 20 cases sequenced at Foundation Medicine. NA when not reported as being performed.
