# Supplementary material for: A Case Series of Metastatic Malignant Gastrointestinal Neuroectodermal Tumors and Comprehensive Genomic Profiling Analysis of 20 Cases
Source: Curr Oncol. 2022 Feb 21;29(2):1279–97. doi: 10.3390/curroncol29020109 (PMC8870546; doi:10.3390/curroncol29020109)
Supplement: Supplementary file 1 [file curroncol-29-00109-s001.zip › curroncol-1534239-supplementary.pdf]

Current Oncology

Supplementary Material

A Case Series of Metastatic Malignant Gastrointestinal Neuroectodermal Tumors and Comprehensive Genomic Profiling Analysis of 20 Cases

Taylor Kandler [1,†], Eliane Cortez [2,†], Lani Clinton [3], Amanda Hemmerich [3], Osama Ahmed [4], Ralph Wong [5], Taylor Forns [6], Andrea J. MacNeill [7], Trevor D. Hamilton [7], Mohammadali Khorasani [7] and Xiaolan Feng [1,8,9,*]

[1] Department of Medicine, University of British Columbia, Vancouver, BC V6T 1Z3, Canada; kandlertaylor@gmail.com
[2] Foundation Medicine, Inc., Cambridge, MA 02141, USA; ecortez@foundationmedicine.com
[3] Foundation Medicine, Inc., Morrisville, NC 27560, USA; lclinton@foundationmedicine.com (L.C.); ahemmerich@foundationmedicine.com (A.H.)
[4] Department of Medical Oncology, Saskatoon Cancer Center, Saskatoon, SK S7N 4H4, Canada; osama.ahmed@saskcancer.ca
[5] Department of Medical Oncology, Cancer Care Manitoba, Manitoba, Winnipeg, MB R3E 0V9, Canada; rwong2@cancercare.mb.ca
[6] Department of Pathology, Duke University, Durham, NC 27710, USA; taylor.forns@duke.edu
[7] Department of Surgery, University of British Columbia, Vancouver, BC V5Z 1M9, Canada; andrea.macneill@bccancer.bc.ca (A.J.M.); trevor.hamilton@vch.ca (T.D.H.); kh.sohrab@gmail.com (M.K.)
[8] Department of Medical Oncology, Tom Baker Cancer Center, Calgary, AB T2N 4N2, Canada
[9] Cumming School of Medicine, University of Calgary, Calgary, AB T2N 4N1, Canada
* Correspondence: fxiaolan@ucalgary.ca
† These authors contributed equally to this work.

Citation: Kandler, T.; Cortez, E.; Clinton, L.; Hemmerich, A.; Ahmed, O.; Wong, R.; Forns, T.; MacNeill, A.J.; Ham-ilton, T.; Khorasani, M.; et al. A Case Series of Metastatic Malignant Gastrointestinal Neuroectodermal Tumors and Comprehensive Genomic Profiling Analysis of 20 Cases. Curr. Oncol. **2022**, *29*, 1279–1297. https://doi.org/10.3390/curroncol29020109

Received: 15 December 2021
Accepted: 14 February 2022
Published: 21 February 2022

Publisher's Note: MDPI stays neutral with regard to jurisdictional claims in published maps and institutional affiliations.

Copyright: © 2022 by the authors. Licensee MDPI, Basel, Switzerland. This article is an open access article distributed under the terms and conditions of the Creative Commons Attribution (CC BY) license (https://creativecommons.org/licenses/by/4.0/).

Table S1. Detailed information of the 20 cases sequenced at Foundation Medicine. NA when not reported as being performed.

Case	Specimen Site	Gender	Age	TMB	MSI Status	IHC					FISH EWSR1	NGS EWSR1 Partner Gene
						HMB-45	MelanA	Melanoma Cocktail	S-100	SOX10		
1	Liver	female	43	1.3	MSS	NA	NA	Negative	Positive	NA	NA	CREB1
2	Small Intestine	female	43	1.3	MSS	Negative	NA	NA	Positive	Positive	Positive	ATF1
3	Small Intestine	female	15	1.3	MSS	NA	NA	Negative	Positive	Negative	Positive	FLI1
4	Small Intestine	female	31	1.6	MSS	Negative	Negative	NA	Positive	Positive	Positive	CREB1
5	Liver	male	61	0	MSS	Negative	NA	NA	Positive	Positive	Positive	CREB1
6	Small Intestine	female	64	1.2	Not Performed	NA	Negative	Negative	Positive	NA	Positive	CREB1
7	Liver	female	48	3.5	MSS	NA	NA	NA	Borderline	Positive	NA	ATF1
8	Small Intestine	female	24	0.9	MSS	Negative	Negative	NA	Positive	NA	Positive	Not identified
9	Small Intestine	male	37	0.8	MSS	NA	NA	NA	NA	NA	NA	CREB1
10	Small Intestine	male	18	4	MSS	NA	Negative	NA	Positive	NA	Positive	ATF1
11	Liver	female	63	0.8	MSS	NA	NA	NA	NA	NA	Positive	CREB1
12	Stomach	female	45	1.6	MSS	NA	NA	NA	NA	NA	NA	ATF1
13	Appendix	male	29	0.8	MSS	Negative	NA	NA	Positive	NA	Positive	ATF1
14	Small Intestine	female	39	0.8	MSS	NA	Negative	NA	Positive	NA	NA	ATF1
15	Soft tissue	male	19	0	MSS	NA	NA	NA	NA	NA	NA	CREB1
16	Abdomen	female	15	0	MSS	Negative	NA	NA	Positive	Positive	NA	ATF1
17	Diaphragm	female	38	0	MSS	NA	NA	NA	NA	NA	NA	FLI1
18	Small Intestine	female	15	0.8	MSS	Negative	Negative	NA	Positive	Positive	Positive	ATF1
19	Extrahepatic bile duct	female	35	1.3	MSS	Negative	Negative	NA	Positive	Positive	Positive	ATF1
20	Chest wall	male	51	1.6	MSS	NA	NA	NA	NA	Positive	NA	ATF1
